# Longitudinal Evaluation of Ataxia and Brain Structural Changes in *RFC1* ‐Related Disorder

**DOI:** 10.1002/mdc3.70344

**Published:** 2025-09-04

**Authors:** Camila C. Lobo, Thiago J.R. Rezende, Luciana R. Pimentel‐Silva, Gustavo M. Jarola, Nadson B.S. Santos, Gabriel da Silva Schmitt, Paula C.A.A.P. Matos, Fabrício D. Lima, Alberto R.M. Martinez, Orlando G.P. Barsottini, José Luiz Pedroso, Wilson Marques, Marcondes C. França

**Affiliations:** ^1^ Department of Neurology School of Medical Sciences – University of Campinas (UNICAMP) Campinas Brazil; ^2^ Department of Neurology, General Neurology and Ataxia Unit Federal University of Sao Paulo (UNIFESP) São Paulo Brazil; ^3^ Department of Neurosciences School of Medicine – University of São Paulo at Ribeirão Preto (USP‐RP) Ribeirão Preto Brazil

**Keywords:** ataxia, MRI, RFC1 expansion

## Abstract

**Background:**

The progression of brain damage in CANVAS/RFC1 remains unclear.

**Objective:**

To describe longitudinal brain changes in CANVAS/RFC1.

**Methods:**

Ten RFC1‐positive patients and 10 controls underwent 3T‐MRI scans 2 years apart. We analyzed cerebral gray and white matter using FreeSurfer and DTI multiatlas, the cerebellum with CerebNet, and the spinal cord with Spinal Cord Toolbox, respectively. Ataxia severity was measured with SARA. Longitudinal changes were assessed using mixed‐effect models and Standardized Response Mean (SRM).

**Results:**

CANVAS/RFC1 patients (mean disease duration: 8.5 ± 3.7 years) showed progressive atrophy in the brainstem (SRM:‐3.5), left hippocampus (SRM:‐2.1), right thalamus (SRM:‐2.6), left cerebellar lobules VI (SRM:‐1.1) and X (SRM:‐0.7), and reduced spinal cord area at C1 and C2 (SRM:‐0.7 each). SARA variation showed lower sensitivity (SRM:0.7).

**Conclusions:**

Patients with CANVAS/RFC1 showed progressive, asymmetrical volume reduction of structures in the brain and spinal cord (C1 and C2), over a period of 2 years, using 3‐tesla MRI.

Biallelic AAGGG intronic expansions in the *RFC1* gene were described in 2019 as the cause of cerebellar ataxia, sensory neuronopathy, and vestibular areflexia syndrome (CANVAS).^1^ Since then, studies have described a broadening of the phenotypic spectrum.^2^, ^3^, ^4^ Considering that not all patients with the biallelic *RFC1* repeat expansions present with the CANVAS triad, the term CANVAS/RFC1 would be more appropriate to define this disease.^5^

In previous studies using advanced neuroimaging, specific structural brain alterations associated with CANVAS/RFC1 have been found, involving not only the cerebellum, but also the deep cerebral gray matter and some white matter tracts.^6^ However, there are no longitudinal assessments to track the progression of these structural changes over time to date.

Therefore, the present study aims to fill this gap by employing longitudinal neuroimaging assessment to characterize alterations in brain as well as spinal cord structure over a 2‐year interval period in patients diagnosed with CANVAS/RFC1. As a secondary objective, our aim is to determine whether these biomarkers are more sensitive than clinical parameters in capturing disease progression.

## Methods

### Subjects

We enrolled 10 RFC1‐positive patients and 10 age‐ and sex‐matched healthy controls. A flowchart of the study design is available in Figure S1 (supplementary material). The patients are followed at the Neurogenetics Outpatient Clinic of the University of Campinas and Federal University of São Paulo. None of the recruited controls had a personal or family history of neurological or psychiatric disorders. Baseline MRI scans were acquired between September 2019 and September 2021 and follow up scans between August 2022 and January 2023. The study protocol was approved by the local Ethics Committee of the University of Campinas under the number CAAE 83241318.3.1001.5404, and written informed consent was obtained from all participants prior to inclusion.

### Clinical Evaluation

All subjects underwent clinical and neurological evaluation, performed by board‐certified neurologists, on the same day MRI scans were obtained. Ataxia severity was quantified using the Brazilian Portuguese validated version of the Scale for Assessment and Rating of Ataxia (SARA).^7^

### MRI Acquisition

All subjects underwent MRI scans on the same 3 T Philips Achieva Scanner (Philips, Best, The Netherlands). A detailed description of the MRI acquisition parameters is described in the supplementary material.

### Image Processing

Based on a hypothesis‐driven approach, data analyses focused on regions known to be involved in the pathophysiology of the disease (Table S1, supplementary information).^1^, ^2^, ^6^, ^8^, ^9^, ^10^ Therefore, gray matter volumetry targeted the brainstem, basal ganglia, diencephalon and hippocampus, while white matter was assessed in the cerebellar peduncles. All gray and white matter structures of the cerebellum were analyzed. Regarding the spinal cord, cross‐sectional area and eccentricity of the first three spinal segments were examined, as the acquired images were brain MRI scans.

We analyzed deep gray matter volumes using FreeSurfer,^11^, ^12^ while cerebellar substructures were quantitatively assessed with CerebNet,^13^ a deep‐learning segmentation tool. White matter microstructure was evaluated with the DTI Multiatlas pipeline,^14^ and spinal cord morphology (eccentricity and cross‐sectional area) was assessed using the Spinal Cord Toolbox (SCT).^15^ All segmentations were visually inspected for accuracy to ensure the reliability of the automated methods and to identify any potential outliers. Further details on these methods are provided in the Supplementary Material.

### Statistics

The descriptive statistics data was presented as mean and standard deviation. Mean age and sex proportions were compared between groups using Student's *t*‐test and Fisher's exact test respectively.

For the longitudinal analysis, we built generalized linear mixed effect models, including group (between‐subjects), MRI follow‐up (within‐subjects) and a group‐by‐MRI follow‐up interaction term as fixed effects, covarying for age, sex, and eTIV. Significant interactions were followed by simple effect analysis. We also included subjects as a random effect to improve generalization of the findings. The models converged to the best fit with linear relationships and using variance component and identity covariance structures for the random and longitudinal effects, respectively. No serious violations of data or models’ assumptions were found. Standardized Response Mean (SRM) was employed to assess sensitivity for longitudinal changes. We used Pearson correlation coefficients to investigate associations between clinical and MRI‐based parameters with FDR adjustment to account for multiple comparisons. The significance level was established at 0.05. For all analyses, we used SPSS v. 29 (IBM corp.©) and MATLAB v. 2022b.

## Results

### Demographic and Clinical Data

CANVAS/RFC1 cohort and healthy controls characteristics are detailed in Table 1. Demographic variables were similar between groups, with no significant differences in age (RFC1: 58.1 ± 6.0 years, Controls: 53.3 ± 7.4 years, *P* = 0.128), sex distribution (RFC1: 60% male, Control: 50% male, *P* = 1.0) and MRI scan interval (RFC1: 2.0 ± 0.6 years, Controls: 2.0 ± 0.9 years, *P* = 0.977).

**TABLE 1:** Demographic and clinical data of CANVAS/*RFC1* cohort and healthy controls

		Age (years)	Sex	Disease duration (baseline)	Follow‐up interval (years)	SARA at baseline	SARA at follow‐up	Cerebellar syndrome	Sensory neuropathy	Vestibular arreflexia	Chronic cough	Dysautonomia	Parkinsonism
*RFC1*	Pt. 01	53	M	13	3.3	24	25	+	+	+	+	+	−
	Pt. 02	56	F	8	1.3	19	22	+	+	+	+	+	−
	Pt. 03	60	M	7	2.1	14.5	26	+	+	+	+	+	−
	Pt. 04	49	M	7	2.2	21.5	31	+	+	+	+	+	+
	Pt. 05	58	M	8	1.3	15.5	16.5	+	+	+	+	+	+
	Pt. 06	68	F	6	2.0	9.5	11.5	+	+	+	+	+	−
	Pt. 07	63	F	16	1.7	14	17.5	+	+	+	+	+	−
	Pt. 08	51	M	5	2.1	14.5	9	−	+	−	−	+	−
	Pt. 09	64	F	11	2.4	12	26.5	+	+	+	+	+	+
	Pt. 10	59	M	4	1.3	7.5	8.5	+	+	+	+	−	−
Healthy controls	Ctl. 01	62	M	−	2.9	−	−	−	−	−	−	−	−
	Ctl. 02	59	M	−	1.2	−	−	−	−	−	−	−	−
	Ctl. 03	44	F	−	2.7	−	−	−	−	−	−	−	−
	Ctl. 04	53	M	−	1.0	−	−	−	−	−	−	−	−
	Ctl. 05	42	F	−	0.7	−	−	−	−	−	−	−	−
	Ctl. 06	48	M	−	2.0	−	−	−	−	−	−	−	−
	Ctl. 07	62	F	−	2.0	−	−	−	−	−	−	−	−
	Ctl. 08	48	M	−	1.2	−	−	−	−	−	−	−	−
	Ctl. 09	57	F	−	3.0	−	−	−	−	−	−	−	−
	Ctl. 10	58	F	−	2.9	−	−	−	−	−	−	−	−

Abbreviation: SARA, Scale of Assessment and Rating of Ataxia.

In the *RFC1* cohort, the mean disease duration was 8.5 ± 3.7 years. The mean SARA score was 15.2 ± 5.1 at baseline, which increased to a mean of 19.4 ± 7.9 at longitudinal assessment. This change corresponds to a SRM of 0.7 (Table S6 supplementary material). Most patients (90%) exhibited the complete CANVAS triad, except for one patient who had pure sensory neuronopathy (Table 1, Pt. 08). Notably, this patient with isolated sensory ataxia showed a reduction in their SARA score over time, reflecting functional improvement following regular physiotherapy.

### Neuroimaging Data

The follow‐up analyses showed that changes over time were a feature of the CANVAS/RFC1 group only, namely: in the deep gray matter, progressive atrophy of the brainstem (*P* = 0.029), left hippocampus (*P* = 0.027), and right thalamus (*P* = 0.044), for the spinal cross‐sectional areas, a significant reduction at C1 (*P* = 0.042) and C2 (*P* = 0.04) levels was also observed. Neuroimaging parameters with significant longitudinal changes in CANVAS/RFC1 vs healthy controls are graphically represented in Figure S2 (supplementary material).

In the cerebellum, although we found significant interactions in the left lobule VI (*P* = 0.027) and the left lobule X (*P* = 0.049), the analysis of simple effects revealed no significant differences for the CANVAS/RFC1 (*P* = 0.062 and *P* = 0.25 respectively). No significant changes were observed in other regions of the cerebellum compared to the changes in the controls. Microstructural analysis of white matter integrity revealed no significant longitudinal changes between groups. Detailed test statistics for the main effects and interactions are presented in Table S2 to S5 (supplementary material).

### SRM of Clinical and Neuroimaging Parameters

**Fig 1:**
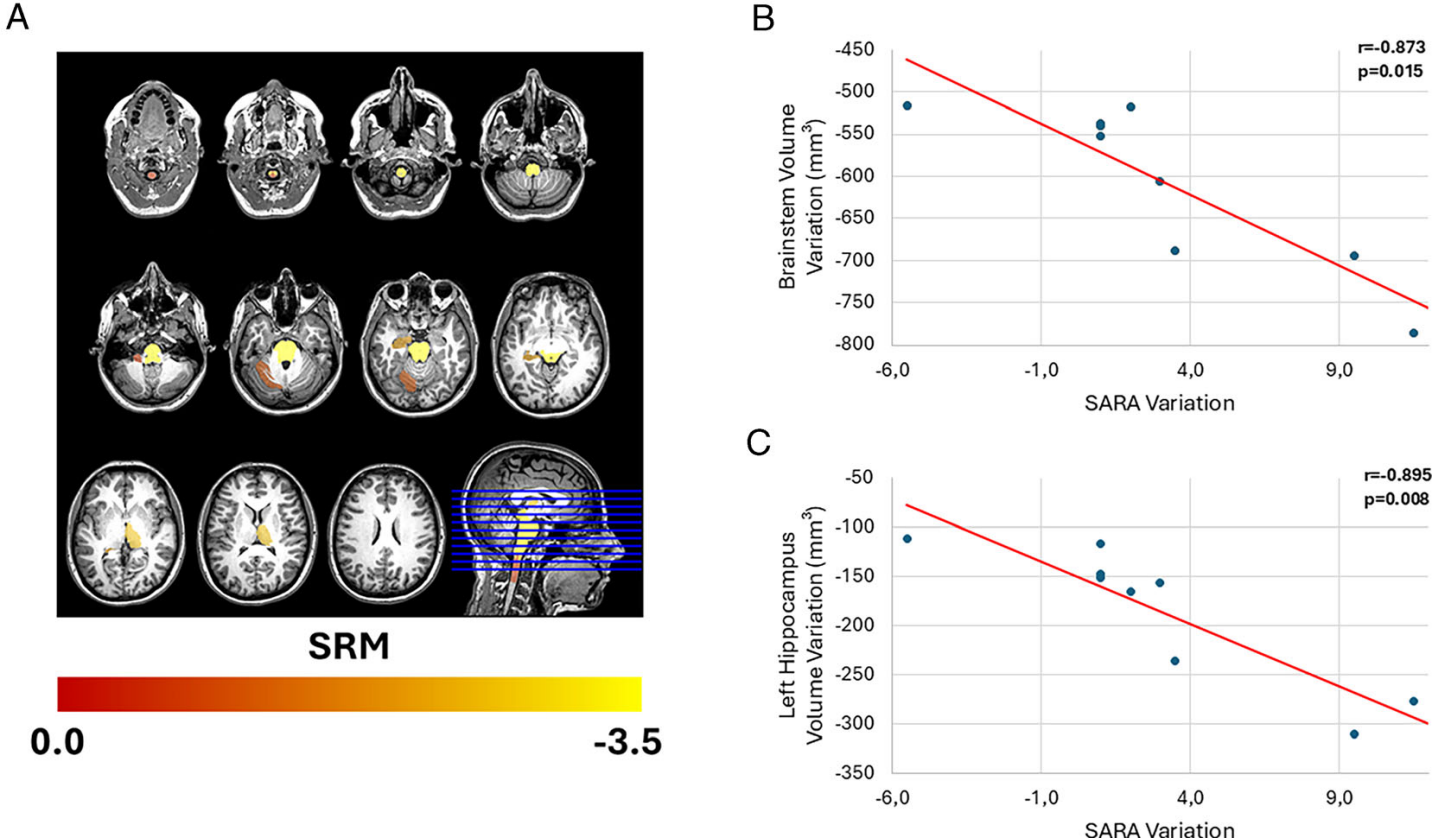
Regions with longitudinal structural change in the RFC1 group relative to controls (using Freesurfer, CerebNet and Spinal Cord Toolbox). The color bar represents magnitude of the standardized response means (SRM) (A). Correlations between volumetric reduction at brainstem (B) and left hippocampus (C) and ataxia severity progression.

The calculation of SRM for neuroimaging parameters that showed a significant interaction between group and time revealed that the brainstem was the most sensitive parameter (SRM = −3.5), followed by the right thalamus (SRM = −2.6) and the left hippocampus (SRM = −2.1). The magnitude of the SRM of the significant areas is represented in Figure 1A. Compared to the SARA score (SRM = 0.7), all neuroimaging parameters had equal or higher SRM values (Table S6, supplementary material).

### Clinical and Neuroimaging Parameters Correlation

The correlation analysis revealed a strong inverse association between the progression of SARA scores and the variation of the brainstem (*r* = −0.873, *P* = 0.019) and the left hippocampus (*r* = −0.895, *P* = 0.019) volumes. These correlations are graphically represented in Figure 1B,C. The SARA score variation did not correlate with the progression of other structural changes.

## Discussion

This is the first study to conduct a longitudinal assessment combining both clinical and neuroimaging data in CANVAS/RFC1. Our results indicate that CANVAS/RFC1 is a progressive condition that affects brain regions involved not only in motor control, but also in cognitive functions and sensory processing. Moreover, we provide evidence that the upper spinal cord is progressively affected by this disease.

Our study confirms that some central nervous system structures show significant volumetric decline over time, even within a short monitoring period.^6^, ^10^ The progression of hippocampal, thalamic, and brainstem atrophy at this stage of the disease may be linked to the occurrence of phenomena beyond the classical presentation, such as parkinsonism, dysautonomia, and cognitive impairment.^2^, ^3^, ^16^, ^17^, ^18^ Despite these findings, the limited previous neuropathological studies have not demonstrated significant neuronal loss in these regions, except for the spinal cord and cerebellum.^8^, ^19^ Previous neuroimaging data align with our findings of reductions in spinal cord cross‐sectional areas, supported by histopathological evidence of dorsal column atrophy and pallor, particularly in the cervical region.^8^, ^10^, ^20^

The asymmetry observed in the progression of structural changes is consistent with findings from previous cross‐sectional study using advanced neuroimaging techniques.^6^ The longitudinal structural changes observed in the brainstem, supported by the SRM of −3.5, highlight its sensitivity as a potential biomarker of disease progression. Annual atrophy rates were substantially higher in patients compared to controls, particularly in the brainstem (−227.4 ± 50 mm^3^/year in patients vs. −34.6 ± 144 mm^3^/year in controls), thalamus (−178.8 ± 75 vs. −21.8 ± 57), and hippocampus (−102.8 ± 52 mm^3^/year vs. +26.8 ± 41 mm^3^/year) (Table S7). These findings reinforce the progressive nature of RFC1‐related neurodegeneration and may suggest possible region‐specific vulnerability. Interestingly, the apparent volume increase in the hippocampus among controls may reflect measurement variability or subtle compensatory processes in healthy aging. Moreover, the strong inverse correlation between brainstem volume and SARA score variation underscores its clinical relevance. Notably, the use of brainstem volumetry as a biomarker is advantageous due to its reliance on a widely available imaging modality commonly employed in the follow‐up of these patients. The use of T1‐weighted MRI also facilitates harmonization across multicenter studies and makes it a feasible potential monitoring biomarker. Additionally, similar SRM values have been reported for subcortical structures in studies on other late‐onset genetic ataxias, particularly spinocerebellar ataxias, further supporting the utility of this approach.^21^

Despite the short monitoring period, our study has revealed significant findings in structures known to be implicated in the disease pathology. However, our small sample and late‐stage cohort are limitations, which may obscure longitudinal alterations and reduce the detection of changes in regions susceptible to early‐stage degeneration, creating a possible ceiling effect. The lack of assessment of cranial nerve thinning, a specific but still uncertain RFC1‐related neuroimaging feature, represents another limitation, as baseline images were acquired before this finding was reported and did not include appropriate sequences.^22^ Future studies should address this by including larger, multicenter, early‐stage cohorts and employing more detailed neuroimaging parameters. Moreover, the segmentation methods should allow for a more granular analysis of the brainstem to identify specific structures, such as the vestibular nuclei.

In conclusion, our study demonstrates that quantitative neuroimaging captures disease progression more sensitively than clinical evaluation with SARA scores and volumetry of the brainstem emerges as a promising monitoring biomarker in CANVAS/RFC1.

## Author Roles

(1) Research project: A. Conception, B. Organization, C. Execution; (2) Statistical analysis: A. Design, B. Execution, C. Review and critique; (3) Manuscript: A. Writing of the first draft, B. Review and critique.

C.C.L.: 1, 2A, 2B, 3A.

T.J.R.R.: 1, 2A, 2C, 3B.

L.R.P.S.: 1C, 2, 3A.

G.M.J.: 1B, 2C, 3B.

N.B.S.S.: 1C, 2C, 3B.

G.S.S.: 1C, 2C, 3B.

P.C.A.A.P.M.: 1C, 2C, 3B.

F.D.L.: 1C, 2C, 3B.

A.R.M.M.: 1B, 2C, 3B.

O.G.P.B.: 1A, 1B, 2C, 3B.

J.L.P.: 1A, 1B, 2C, 3B.

W.M.Jr.: 1A, 1B, 2C, 3B.

M.C.F.Jr.: 1, 2A, 2C, 3B.

## Disclosures

**Ethical Compliance Statement**: The study protocol was approved by the local Ethics Committee (Comitê de Ética e Pesquisa—UNICAMP) under the number CAAE 83241318.3.1001.5404 and written informed consent was obtained from all participants before inclusion. We confirm that we have read the Journal's position on issues involved in ethical publication and affirm that this work is consistent with those guidelines.

**Funding Sources and Conflict of Interest**: Fundação de Amparo à Pesquisa do Estado de São Paulo—FAPESP, São Paulo, Brazil—provides support for Dr JLP, OGPB, WMJr, and MCFJr, alongside Conselho Nacional de Pesquisa (CNPq)—Brazil. Additionally, Dr TJRR and MCFJ received funding from the Friedreich's Ataxia Research Alliance (FARA). The funding agencies did not influence the study's design, data collection, or manuscript drafting process. The remaining authors have no additional declarations to disclose.

**Financial Disclosures for the previous 12 months**: CCL: none. TJRR: none. LRPS: none. GMJ: none. NBSS: none. GSS: none. PCAAPM: none. FDL: none. ARMM: none. JLP: none. OGPB: none. WMJr: none. MCF: none. The authors declare that there are no additional disclosures to report.

## Supporting information


**Figure S1.** Study design.
**TABLE S1.** References for hypothesis‐driven selection of anatomical structures in longitudinal neuroimaging of RFC1‐related disorder.
**TABLE S2.** Mean volume at baseline and follow‐up scans and statistics longitudinal changes of deep gray matter volumetry.
**TABLE S3.** Mean volume at baseline and follow‐up scans and statistics longitudinal changes of cerebellar gray and white matter volumetry.
**TABLE S4.** Mean volume at baseline and follow‐up scans and statistics longitudinal changes of quantitative spinal cord morphometry.
**TABLE S5.** Mean volume at baseline and follow‐up scans and statistics longitudinal changes of microstructural analysis of white matter integrity.
**TABLE S6.** Standardized response means of clinical and neuroimaging changes in the *RFC1* cohort.
**TABLE S7.** Annualized Volumetric Changes in Brain Regions for RFC1 and Healthy Controls.
**Figure S2.** Neuroimaging parameters with significant longitudinal changes in RFC1‐related disorders vs healthy controls. A, brainstem volumetry. B, right thalamus volumetry. A, left hippocampus volumetry. D, spinal cord cross‐sectional area at C1 level. E, spinal cord cross‐sectional area at C2 level. F, left cerebellar VI lobule volumetry. G, left X cerebellar lobule volumetry. Asterisk indicates significant results in the analysis of simple effects.

## Data Availability

The data that support the findings of this study are available on request from the corresponding author. The data are not publicly available due to privacy or ethical restrictions.
