# Supplementary material for: Longitudinal Evaluation of Ataxia and Brain Structural Changes in RFC1 ‐Related Disorder
Source: Mov Disord Clin Pract. 2025 Sep 4;12(12):2311–6. doi: 10.1002/mdc3.70344 (PMC12715363; doi:10.1002/mdc3.70344)
Supplement: Supplementary file 1 — Figure S1. Study design. TABLE S1. References for hypothesis‐driven selection of anatomical structures in longitudinal neuroimaging of RFC1‐related disorder. TABLE S2. Mean volume at baseline and follow‐up scans and statistics longitudinal changes of deep gray matter volumetry. TABLE S3. Mean volume at baseline and follow‐up scans and statistics longitudinal changes of cerebellar gray and white matter volumetry. TABLE S4. Mean volume at baseline and follow‐up scans and statistics longitudinal changes of quantitative spinal cord morphometry. TABLE S5. Mean volume at baseline and follow‐up scans and statistics longitudinal changes of microstructural analysis of white matter integrity. TABLE S6. Standardized response means of clinical and neuroimaging changes in the RFC1 cohort. TABLE S7. Annualized Volumetric Changes in Brain Regions for RFC1 and Healthy Controls. Figure S2. Neuroimaging parameters with significant longitudinal changes in RFC1‐related disorders vs healthy controls. A, brainstem volumetry. B, right thalamus volumetry. A, left hippocampus volumetry. D, spinal cord cross‐sectional area at C1 level. E, spinal cord cross‐sectional area at C2 level. F, left cerebellar VI lobule volumetry. G, left X cerebellar lobule volumetry. Asterisk indicates significant results in the analysis of simple effects. [file MDC3-12-2311-s001.docx]

**SUPPLEMENTARY DATA**

**Magnetic Resonance Imaging (MRI) Acquisition**

All participants underwent MRI scanning on a 3T Philips Achieva Scanner with a standard 8-channel head coil. Additionally, routine T2-weighted images were obtained to rule out any unrelated abnormalities, such as white matter diseases and minor strokes, which were thoroughly reviewed by an experienced neuroradiologist.

For volumetry and morphometry analyses, high-resolution T1 volumetric brain images with sagittal orientation were utilized. These images had a voxel matrix of 240×240×180, voxel size of 1×1×1 mm³, TR/TE of 7/3.201 ms, and a flip angle of 8°. The T1 sequence used was a 3D-SPGR acquisition. The scanning duration was ten minutes.

A spin echo diffusion tensor imaging (DTI) sequence was employed for DTI multi-atlas analyses. The acquisition voxel size was 2×2×2 mm³, interpolated to 1×1×2 mm³, with a reconstructed matrix of 256×256, 70 slices, TE/TR of 61/8500 ms, flip angle of 90°, 32 gradient directions, no averages, and a maximum b-factor of 1000 s/mm². The scanning duration for this sequence was six minutes.

**DTI multi-atlas**

To evaluate white matter abnormalities, we utilized the "MRICloud" (MRICloud.org), a publicly available web-based platform for multi-contrast imaging segmentation and quantification. Initially, raw DTI-weighted images were subjected to co-registration and correction for eddy currents, as well as correction for subject motion using a 12-parameter affine transform.^1-3^ Subsequently, DTI parameters were computed via multivariate linear fitting employing DTIStudio software (H. Jiang and S. Mori, Johns Hopkins University, Kennedy Krieger Institute).^4^ Skull-stripping was then conducted through intensity thresholding using the b=0 image, employing the RoiEditor tool within the software suite (Li, X.; Jiang, H.; Yue, Li.; and Mori, S.; Johns Hopkins University, www.MriStudio.org or www.kennedykrieger.org). Following this step, a non-linear registration utilizing multi-contrast LDDMM was executed to enhance alignment and correspondence between the atlas and the processed image.^5^ White matter parcellation was performed utilizing the Diffeomorphic Likelihood Fusion Algorithm (DLFA).^5^ DLFA employs Bayesian estimators to model the intensity distribution for each label, incorporating various types of information including anatomical position within the brain, fractional anisotropy, medial diffusivity, fiber angle, and primary eigenvector.^5^ A total of eight atlases (JHU adult atlas version 1) were employed to generate 168 structures. From these structures, the corticospinal and peduncular tracts were selected for longitudinal evaluation. All analyses were conducted in native space and computed on the Gordon cluster of XSEDE.^6^ DTI multi-atlas analysis provides metrics of diffusivity such as fractional anisotropy (FA), medial diffusivity (MD), axial diffusivity (AD), and radial diffusivity (RD).

**Freesurfer**

The thickness measurements were obtained using FreeSurfer software (version 6.0) following protocols proposed by Fischl and Dale.^7^ Subcortical, cortical gray matter, and cerebrospinal fluid (CSF) voxels were segmented based on their location and intensity. A triangular mesh network was built around the gray matter surface, smoothed considering local intensity using trilinear interpolation. Topological defects are corrected to ensure a sphere-like surface. A second smoothing iteration generates a White Surface, then nudged outwards to create the pial surface. This surface is segmented into neuroanatomical regions using automated processes. The pial surface is homeomorphically mapped onto a spherical coordinate system, allowing Bayesian segmentation assigning neuroanatomical labels to each vertex. Cortical thickness is calculated as the minimum distance between pial and white surfaces along the cortical mantle. Gaussian smoothing with a FWHM of 10 mm and subject-wise averaging were applied to all maps for analyses. Deep GM structures are labeled following the methodology proposed by Fischl and colleagues, considering the intensity histogram of such structures.^7,8^

**Spinal Cord Toolbox**

We employed the segmentation method provided by the Spinal Cord Toolbox (SCT) to conduct morphometric analysis of the spinal cord. Initially, we applied the “propagation of labels” technique implemented in the SCT to delineate the spinal cord from surrounding tissues.^9^ This involved a combination of manual intervention and automated processes to accurately define the boundaries of the spinal cord.^10^ Subsequently, we utilized an active contours algorithm within the SCT to further refine the segmentation boundaries, ensuring precise delineation of the spinal cord structure.^9^ Additionally, we incorporated advanced techniques, such as multi-atlas-based segmentation, to enhance the accuracy of our analysis by leveraging information from multiple spinal cord atlases.^11^ Following segmentation, we conducted rigorous quality control checks, including visual inspection and statistical analysis, to validate the accuracy of our morphometric measurements.

**CerebNet**

Faber et al. introduced CerebNet as an advanced cerebellar segmentation algorithm, building upon the Fastsurfer framework.^12^ Employing a U-Net architecture with three convolutional neural networks, each dedicated to a specific axis, the algorithm combines label probability evaluations from axial, sagittal, and coronal orientations, standing out as the first algorithm evaluated using clinical population data.^12^ This showcases its ability to capture detailed boundaries between cerebellar white and gray matter with high precision. We utilized CerebNet to perform sub-segmentation of the cerebellum into the following regions: the cerebellar vermis and its subdivisions, the white matter of each cerebellar hemisphere separately, and the gray matter of each cerebellar hemisphere collectively, according to the subdivisions of the lobules. All scans underwent visual inspection. To adjust for individual differences in head size, we utilized the estimated total intracranial volume (eTIV).

**References**

1. Andersson JLR, Skare S. A model-based method for retrospective correction of geometric distortions in diffusion-weighted EPI. Neuroimage. 2002 May;16(1):177–99.

2. Zhuang J, Hrabe J, Kangarlu A, Xu D, Bansal R, Branch CA, et al. Correction of eddy-current distortions in diffusion tensor images using the known directions and strengths of diffusion gradients. J Magn Reson Imaging. 2006 Nov;24(5):1188–93.

3. Woods RP, Grafton ST, Holmes CJ, Cherry SR, Mazziotta JC. Automated image registration: I. General methods and intrasubject, intramodality validation. J Comput Assist Tomogr. 1998 Jan-Feb;22(1):139–52.

4. DtiStudio: Resource program for diffusion tensor computation and fiber bundle tracking. Comput Methods Programs Biomed. 2006 Feb 1;81(2):106–16.

5. Tang X, Yoshida S, Hsu J, Huisman TAGM, Faria AV, Oishi K, et al. Multi-contrast multi-atlas parcellation of diffusion tensor imaging of the human brain. PLoS One. 2014 May 8;9(5):e96985.

6. Towns J, Cockerill T, Dahan M, Foster I, Gaither K, Grimshaw A, et al. XSEDE: Accelerating scientific discovery. Comput Sci Eng. 2014 Sep;16(5):62–74.

7. Fischl B, Dale AM. Measuring the thickness of the human cerebral cortex from magnetic resonance images. Proc Natl Acad Sci U S A. 2000 Sep 26;97(20):11050–5.

8. Fischl B, Salat DH, Busa E, Albert M, Dieterich M, Haselgrove C, et al. Whole brain segmentation: automated labeling of neuroanatomical structures in the human brain. Neuron. 2002 Jan 31;33(3):341–55.

9. De Leener B, Lévy S, Dupont SM, Fonov VS, Stikov N, Louis CD, et al. SCT: Spinal Cord Toolbox, an open-source software for processing spinal cord MRI data. Neuroimage [Internet]. 2017 Jan 15 [cited 2024 Mar 20];145(Pt A). Available from: https://pubmed.ncbi.nlm.nih.gov/27720818/

10. Automatic segmentation of the spinal cord and intramedullary multiple sclerosis lesions with convolutional neural networks. Neuroimage. 2019 Jan 1;184:901–15.

11. PAM50: Unbiased multimodal template of the brainstem and spinal cord aligned with the ICBM152 space. Neuroimage. 2018 Jan 15;165:170–9.

12. Faber J, Kügler D, Bahrami E, Heinz LS, Timmann D, Ernst TM, et al. CerebNet: A fast and reliable deep-learning pipeline for detailed cerebellum sub-segmentation. Neuroimage. 2022 Dec 1;264:119703.

**Figure S1**. Study design.


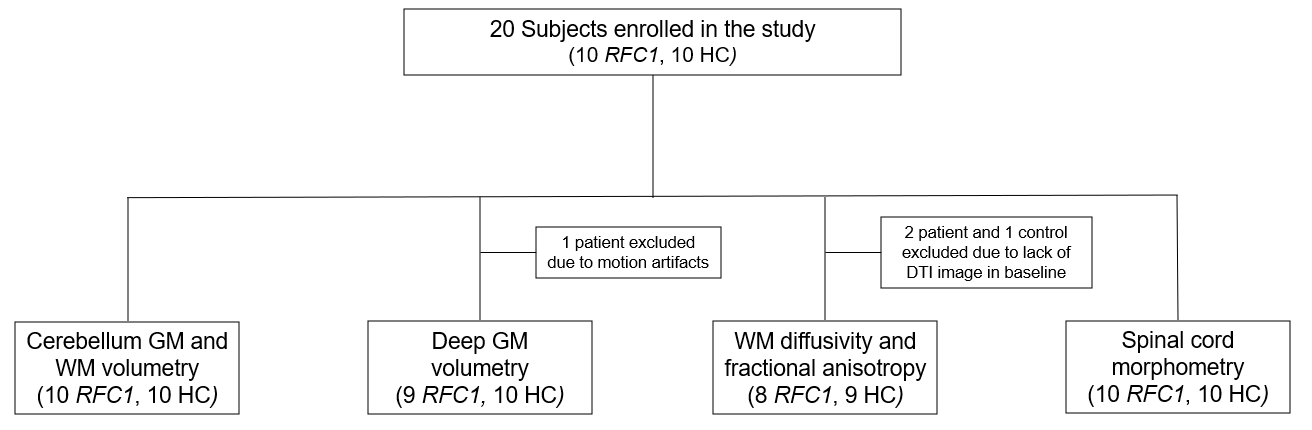


Abbreviations: HC, healthy controls; GM, gray matter; WM, white matter.

**Table S1**. References for hypothesis-driven selection of anatomical structures in longitudinal neuroimaging of RFC1-related disorder.

| Structures |  | References |
| --- | --- | --- |
| Brainstem | Neuroimaging  Anatomopathological | Traschütz, A. et al. 2021. Matos, PCAAP. et al. 2021.  Huin V. et al. 2022. |
| Hippocampus | Neuroimaging  Anatomopathological | Matos, PCAAP. et al. 2021.  Huin V. et al. 2022. |
| Thalamus | Neuroimaging  Anatomopathological | Matos, PCAAP. et al. 2021.  **-** |
| Pallidum | Neuroimaging  Anatomopathological | Traschütz, A et al. 2021. Matos, PCAAP. et al. 2021.  - |
| Caudate nucleus | Neuroimaging  Anatomopathological | Matos, PCAAP. et al. 2021.  ^-^ |
| Ventral diencephalon | Neuroimaging  Anatomopathological | Matos, PCAAP. et al. 2021.  **-** |
| Amygdala | Neuroimaging  Anatomopathological | Matos, PCAAP. et al. 2021.  Huin V. et al. 2022. |
| Cerebellar peduncles | Neuroimaging  Anatomopathological | Matos, PCAAP. et al. 2021.  Huin V. et al. 2022. |
| Cerebellum | Neuroimaging  Anatomopathological | Traschütz, A et al. 2021. Matos, PCAAP. et al. 2021. Cortese, A. et al. 2019. Cortese, A. et al. 2020.  Huin V. et al. 2022. Cortese, A. et al. 2019. |
| Spinal cord | Neuroimaging  Anatomopathological | Rezende TJR. 2022. Cortese, A. et al. 2020.  Huin V. et al. 2022. |

| Volumetry (mm³) | Mean ±SD | | | | Test statistic  p-values | | |
| --- | --- | --- | --- | --- | --- | --- | --- |
|  | ***RFC1*** | | **HC** | | ***RFC1* vs HC** | | |
|  | **Baseline** | **Follow-up** | **Baseline** | **Follow-up** | **Group effect** | **Scan visit** | **Group*Scan visit** |
| Brainstem | 17526.9 ±721.2 | 17.366.5 ±725.4 | 20732.7 ±687.2 | 20878.7 ±683.1 | F_1,19.2_ = 10.9 *p = 0.004* | F_1,23.9_ = 0.1 p = 0.752 | **F_1,18.9_ = 34.3 *p < 0.001*** |
| Ventral Diencephalon  Right    Left | 3306.2 ±132.4  3171.9 ±124.0 | 3239.14 ±136.6  3107.9 ±127.1 | 4027.5 ±129.4  3994.6 ±120.4 | 4043.2 ±125.4  3976.2 ± 117.5 | F_1,19.3_ = 16.7 *p < 0.001*  F_1,18.6_ = 23.3  *p < 0.001* | F_1,37.9_ = 0.4 p = 0.512  F_1,37.3_ = 1.9  p = 0.175 | F_1,18.9_ = 3.3 p = 0.087  F_1,18.1_ = 2.4  p = 0.137 |
| Thalamus  Right    Left | 6221.2 ±222.1  6228.9 ±228.1 | 6103.5 ±227.2  6168.8 ±235.4 | 7005.1 ±215.2  6868.6 ±223.1 | 7021.7 ±210.4  6960.9 ±216.0 | F_1,18.7_ = 7.4  *p = 0.014*  F_1,19.3_ = 4.9  *p = 0.038* | F_1,36.7_ = 0.9  p = 0.324  F_1,37.9_ = 0.1  p = 0.813 | **F_1,18.3_ = 7.9**  ***p = 0.012***  F_1,18.9_ = 3.6  p = 0.074 |
| Pallidum  Right    Left | 1567.6 ±50.8  1476.9 ±52.3 | 1582 ±52.6  1434.1 ±54.3 | 1813.5 ±49.9  1907.8 ±51.5 | 1860.9 ±48.1  1898.9 ±49.7 | F_1,19.1_ = 13.8  *p < 0.001*  F_1,19.1_ = 36.9  *p < 0.001* | F_1,36.0_ = 2.4  p = 0.129  F_1,37.7_ = 2.1  p = 0.157 | F_1,18.9_ = 1.2  p = 0.287  F_1,18.9_ = 1.9  p = 0.178 |
| Caudate nucleus  Right    Left | 3286.7 ±93.5  3119.1 ±90.6 | 3221.5 ±96.9  2996.2 ±93.9 | 3675.7 ±91.8  3522.8 ±89.1 | 3683.2 ±88.5  3500.3 ±85.9 | F_1,19.2_ = 10.6  *p = 0.004*  F_1,19.1_ = 13.1  *p = 0.002* | F_1,36.9_ = 0.7  p = 0.412  F_1,34.7_ = 3.7  p = 0.063 | F_1,19.0_ = 2.1  p = 0.168  F_1,18.9_ = 2.8  p = 0.109 |
| Hippocampus  Right    Left | 3830.2 ±99.9  3735.2 ±83.0 | 3843.9 ±102.9  3660.0 ±85.9 | 4101.4 ±97.6  3927.7 ±81.4 | 4174.8 ±94.6  3963.8 ±19.7 | F_1,19.3_ = 4.6  *p = 0.045*  F_1,19.1_ = 4.5  *p = 0.047* | F_1,37.9_ = 2.3  p = 0.141  F_1,37.9_ = 0.5  p = 0.474 | F_1,18.9_ = 3.2  p = 0.092  **F_1,18.9_ = 10.2**  ***p = 0.005*** |
| Amygdala  Right    Left | 1913.3 ±49.0  1643.2 ±61.8 | 1907.8 ±50.8  1669.7 ±58.5 | 2027.7 ±48.2  1880.9 ±60.4 | 2064.9 ±46.4  1901.6 ±58.5 | F_1,18.9_ = 3.9  p = 0.061  F_1,19.2_ = 7.3  *p = 0.014* | F_1,35.9_ = 0.7  p = 0.415  F_1,37.9_ = 1.6  p = 0.207 | F_1,18.9_ = 2.1  p = 0.161  F_1,18.9_ = 0.1  p = 0.792 |

**Table S2**. Mean volume at baseline and follow-up scans and statistics longitudinal changes of deep gray matter volumetry.

**Table S3.** Mean volume at baseline and follow-up scans and statistics longitudinal changes of cerebellar gray and white matter volumetry.

| Volumetry (mm³) | Mean ±SD | | | | Test statistic  p-values | | |
| --- | --- | --- | --- | --- | --- | --- | --- |
|  | ***RFC1*** | | **HC** | | ***RFC1* x HC** | | |
|  | **Baseline** | **Follow-up** | **Baseline** | **Follow-up** | **Group effect** | **Scan visit** | **Group*Scan visit** |
| Vermis  VI  VII  VIII  IX  X | 3372.7 ±291.2  947.3 ±102.4  398.2 ±45  1231.6 ±103.4  584.9 ±52.3  215.8 ±17.5 | 3236.0 ±302.4  913.9 ±106.3  371.7 ±47.1  1192 ±107  555.9 ±54.7  214.6 ±18.4 | 4509.6 ±302.3  1297.7 ±106.2  515.8 ±47.1  1576.5 ±107  746.1 ±54.6  361.4 ±18.4 | 4440.6 ±291.3  1310 ±102.4  498.7 ±45  1528.6 ±103.4  742.6 ±52.3  354.9 ±17.5 | F_1,21.1_ = 7.5  *p = 0.012*  F_1,21.1_ = 6.2  *p = 0.021*  F_1,20.0_ = 3.4  *p = 0.079*  F_1,21.2_ = 5.1  *p = 0.035*  F_1,20.7_ = 5.1  *p = 0.034*  F_1,20.3_ = 31.2  *p = <0.001* | F_1,39.9_ = 2.8  p = 0.105  F_1,39.9_ = 0.2  p = 0.636  F_1,39.6_ = 3.2  p = 0.083  F_1,39.8_ = 4.5  p = 0.039  F_1,39.9_ = 1.5  p = 0.233  F_1,35.9_ = 0.4  p = 0.526 | F_1,19.9_ = 1.0  p = 0.326  F_1,19.9_ = 4.1  p = 0.056  F_1,19.4_ = 0.3  p = 0.582  F_1,19.9_ = 0.2  p = 0.693  F_1,20_ = 2.1  p = 0.159  F_1,19.9_ = 0.3  p = 0.588 |
| White Matter  Right  Left | ­  10079.7 ±620.2  10106.8 ±650.8 | 9871.7 ±632.8  9816.4 ±669.8 | 12526.2 ±632.7  12578.6 ±669.7 | 12430.4 ±620.2  12446.9 ±650.9 | F_1,20.5_ = 7.8  *p = 0.011*  F_1,20.7_ = 7.3  *p = 0.014* | F_1,34.1_ = 2.9  p = 0.094  F_1,38.7_ = 3.4  p = 0.072 | F_1,19.3_ = 2.4  p = 0.136­  F_1,19.4_ = 2.3  p = 0.146 |
| Cortex  Right  Left | 31100.5 ±2578.5  30619.6 ±2773.7 | 30632.3 ±2642.3  29607.2 ±2848.5 | 39795.9 ±2641.9  41020 ±2847.9 | 39699.8 ±2578.7  40551.9 ±2773.9 | F_1,21.3_ = 5.7  *p = 0.027*  F_1,21.3_ = 7.0  *p = 0.015* | F_1,36.9_ = 0.5  p = 0.495  F_1,37.9_ = 2.6  p = 0.118 | F_1,19.9_ = 1.1  p = 0.305  F_1,19.9_ = 1.7  p = 0.202 |
| Crus I  Right  Left | 7045.4 ±611.3  6826.6 ±626.1 | 6918.3 ±629.7  6495.8 ±649.1 | 9156.5 ±629.6  9116.2 ±648.9 | 9068.9 ±611.4  8997.5 ±626.2 | F_1,20.9_ = 5.7  *p = 0.026*  F_1,21.1_ = 6.8  *p = 0.016* | F_1,38.9_ = 0.9  p = 0.331  F_1,39.9_ = 3.0  p = 0.088 | F_1,19.6_ = 0.2  p = 0.702  F_1,19.9_ = 2.5  p = 0.132 |
| Crus II  Right  Left | 4413.8 ±455.7  4135.8 ±462.4 | 4431.9 ±474.9  4124.8 ±478.4 | 5425.8 ±474.8  5282.7 ±478.3 | 5434 ±455.7  5248.4 ±462.5 | F_1,20.9_ = 2.3  p = 0.147  F_1,20.8_ = 2.8  p = 0.108 | F_1,39.9_ < 0.1  p = 0.9  F_1,39.8_ = 0.1  p = 0.804 | F_1,19.9_ = < 0.1  p = 0.943  F_1,19.6_ = 0.1  p = 0.802 |
| Lobule IV  Right  Left | 2037.4 ±116.7  2074.3 ±129.3 | 2015.0 ±121.4  2004.4 ±133.5 | 2745.5 ±121.4  2844.2 ±133.4 | 2733.8 ±116.7  2803.8 ±129.3 | F_1,20.4_ = 17.4  *p < 0.001*  F_1,21.1_ = 17.3  *p < 0.001* | F_1,39.9_ = 0.4  p = 0.515  F_1,39.5_ = 5.2  *p = 0.028* | F_1,19.4_ = 0.1  p = 0.718  F_1,19.8_ = 1.6  p = 0.224 |
| Lobule V  Right  Left | 2119.3 ±174.9  2159.4 ±168.7 | 2050.3 ±182.8  2077.6 ±176.2 | 2995.6 ±182.7  2881.4 ±176.1 | 2976.7 ±174.9  2841.9 ±168.7 | F_1,20.8_ = 12.3  *p = 0.002*  F_1,20.2_ = 8.9  *p = 0.007* | F_1,39.9_ = 0.9  p = 0.332  F_1,39.9_ = 2.1  p = 0.157 | F_1,19.9_ = 0.7  p = 0.401  F_1,19.4_ = 0.6  p = 0.437 |
| Lobule VI  Right  Left | 4802.5 ±482.6  4769.5 ±482.6 | 4598.5 ±496.3  4616.8 ±493.5 | 6137.1 ±496.2  6256.4 ±493.4 | 6046.2 ±482.6  6249.7 ±482.6 | F_1,21.2_ = 3.9  p = 0.060  F_1,20.9_ = 5.0  *p = 0.036* | F_1,38.4_ = 3.2  p = 0.084  F_1,35.7_ = 1.2  p = 0.280 | F_1,19.8_ = 2.2  p = 0.150  **F_1,19.6_ = 5.7**  ***p = 0.027*** |
| Lobule VIIb  Right  Left | 2831.5 ±277.2  2975.5 ±349.9 | 2808.6 ±290.1  2912.8 ±364.8 | 3635.2 ±290.1  4085.9 ±364.7 | 3639.9 ±277.2  4067.3 ±349.9 | F_1,20.5_ = 4.0  p = 0.058  F_1,20.9_ = 4.9  *p = 0.039* | F_1,39.6_ = 0.01  p = 0.905  F_1,39.9_ = 0.2  p = 0.624 | F_1,19.9_ = 0.07  p = 0.793  F_1,199._ = 0.2  p = 0.660 |
| Lobule VIIIa  Right  Left | 3301.2 ±310.4  3337.2 ±348.9 | 3278.1 ±317.4  3180.9 ±363.7 | 3884.4 ±322.6  4672.9 ±363.6 | 3963.6 ±304.9  4579.4 ±349 | F_1,21.2_ = 1.9  p = 0.174  F_1,20.5_ = 7.1  *p = 0.015* | F_1,39.9_ = 0.2  p = 0.691  F_1,39.9_ = 2.3  p = 0.134 | F_1,19.9_ = 1.5  p = 0.228  F_1,19.6_ = 0.4  p = 0.527 |
| Lobule VIIIb  Right  Left | 2495.2 ±209  2361.3 ±220 | 2499.3 ±216.6  2354.4 ±229.1 | 3010.9 ±216.5  2892 ±228.9 | 3049.3 ±20.1  2891.6 ±220.1 | F_1,20.9_ = 3.0  p = 0.096  F_1,20.9_ = 2.7  p = 0.113 | F_1,39.9_ = 0.3  p = 0.619  F_1,40_ = 0.01  p = 0.942 | F_1,19.7_ = 0.6  p = 0.446  F_1,19.9_ = 0.01  p = 0.911 |
| Lobule IX  Right  Left | 1799.6 ±190.6  1799.9 ±191 | 1751.9 ±194.7  1792.9 ±196.3 | 2412.4 ±194.7  2325 ±196.3 | 2366 ±190.6  2320.1 ±191.1 | F_1,21.1_ = 4.9  *p = 0.037*  F_1,20.9_ = 3.6  p = 0.072 | F_1,35.1_ = 2.8  p = 0.102  F_1,38.1_ = 0.03  p = 0.856 | F_1,19.8_ = 0.003  p = 0.968  F_1,19.6_ = 0.006  p = 0.940 |
| Lobule X  Right  Left | 242.8 ±34.4  266.9 ±35.9 | 252.1 ±35.6  255.6 ±37.4 | 420.4 ±35.6  455.4 ±37.4 | 432.4 ±34.4  464.6 ±35.9 | F_1,20.2_ = 12.7  *p = 0.002*  F_1,20.2_ = 14.2  *p = 0.001* | F_1,39.7_ = 2.5  p = 0.118  F_1,40_ = 0.02  p = 0.898 | F_1,18.9_ = 0.2  p = 0.684  **F_1,19.2_ = 4.4**  ***p = 0.049*** |

**Table S4**. Mean volume at baseline and follow-up scans and statistics longitudinal changes of quantitative spinal cord morphometry.

| Metric/ROI | Mean ±SD | | | | Test statistic  p-values | | |
| --- | --- | --- | --- | --- | --- | --- | --- |
|  | ***RFC1*** | | **HC** | | ***RFC1* x HC** | | |
|  | **Baseline** | **Follow-up** | **Baseline** | **Follow-up** | **Group effect** | **Scan visit** | **Group*Scan visit** |
| Cross-sectional area (mm²)  C1  C2  C3 | 4.6 ±0.2  4.47 ±0.14  4.46 ±0.15 | 4.4 ±0.2  4.33 ±0.16  4.33 ±0.17 | 6.6 ±0.2  6.47 ±0.15  6.52 ±0.17 | 6.7 ±0.2  6.46 ±0.15  6.48±0.16 | F_1,20.1_ = 70.8  *p < 0.001*  F_1,**_ = 92.6  *p < 0.001*  F_1,21.1_ = 88.6  *p < 0.001* | F_1,37.9_ = 0.05  p = 0.819  F_1,86.3_ = 3.7  p = 0.059  F_1,37.6_ = 3.1  p = 0.089 | **F_1,19.8_ = 7.5**  ***p = 0.013***  **F_1,25.9_ = 6.2**  ***p = 0.020***  F_1,19.6_ = 1.8  p = 0.191 |
| Eccentricity  C1  C2  C3 | 0.793 ±0.016  0.816 ±0.010  0.861 ±0.007 | 0.785 ±0.016  0.814 ±0.012  0.855 ±0.007 | 0.698 ±0.017  0.759 ±0.011  0.805 ±0.008 | 0.696 ±0.015  0.759 ±0.011  0.804 ±0.006 | F_1,20.5_ = 16.5  *p < 0.001*  F_1,20.1_ = 13.2  *p = 0.002*  F_1,20.5_ = 30.6  *p < 0.001* | F_1,37.4_ = 1.3  p = 0.268  F_1,31.1_ = 0.02  p = 0.887  F_1,28.9_ = 1.5  p = 0.226 | F_1,19.9_ = 0.5  p = 0.488  F_1,19.9_ = 0.06  p = 0.807  F_1,19.9_ = 0.9  p = 0.347 |

**Table S5**. Mean volume at baseline and follow-up scans and statistics longitudinal changes of microstructural analysis of white matter integrity.

| Metric/ROI | Mean ±SD | | | | Test statistic  p-values | | |
| --- | --- | --- | --- | --- | --- | --- | --- |
|  | ***RFC1*** | | **HC** | | ***RFC1* x HC** | | |
|  | **Baseline** | **Follow-up** | **Baseline** | **Follow-up** | **Group effect** | **Scan visit** | **Group*Scan visit** |
| AD  Superior Cerebellar Peduncle  Right  Left  Middle Cerebellar Peduncle  Right  Left  Inferior Cerebellar Peduncle  Right  Left | 0.001885  ±0.000027  0.001854  ±0.000031  0.001229  ±0.000024  0.001252  ±0.000027  0.001536  ±0.000039  0.001551  ±0.000027 | 0.001886  ±0.000028  0.001913  ±0.000032  0.001244  ±0.000025  0.001287  ±0.000028  0.001561  ±0.000041  0.001560  ±0.000028 | 0.001752 ±0.000027  0.001689  ±0.000029  0.001093  ±0.000023  0.001125  ±0.000026  0.001356  ±0.000039  0.001395  ±0.000026 | 0.001725  ±0.000026  0.001699  ±0.000029  0.001098  ±0.000022  0.001125  ±0.000025  0.001396  ±0.000037  0.001447  ±0.000025 | F_1,16.9_ = 19.5  *p < 0.001*  F_1,17_ = 21.2  *P < 0.001*  F_1,16.9_ = 18.1  p < 0.001  F_1,16.9_ = 15.1  p < 0.001  F_1,17.1_ = 9.6  p = 0.007  F_1,17_ = 15.4  p < 0.001 | F_1,18.5_ = 0.4  p = 0.546  F_1,20.7_ = 4.3  p = 0.051  F_1,24.4_ = 0.9  p = 0.328  F_1,23.9_ = 2.4  p = 0.136  F_1,27.6_ = 5.6  p = 0.025  F_1,19.5_ = 3.2  p = 0.091 | F_1,16.9_ = 0.5  p = 0.511  F_1,16.9_ = 2.3  p = 0.147  F_1,16.8_ = 0.4  p = 0.539  F_1,16.9_ = 3.1  p = 0.098  F_1,16.9_ = 0.4  p = 0.544  F_1,17_ = 1.7  p = 0.208 |
| FA  Superior Cerebellar Peduncle  Right  Left  Middle Cerebellar Peduncle  Right  Left  Inferior Cerebellar Peduncle  Right  Left | 0.496 ±0.012  0.483 ±0.013  0.489 ±0.011  0.483 ±0.008  0.431 ±0.014  0.470 ±0.012 | 0.497 ±0.013  0.478 ±0.015  0.499 ±0.009  0.494 ±0.008  0.410 ±0.013  0.441 ±0.015 | 0.516 ±0.012  0.519 ±0.013  0.504 ±0.010  0.513 ±0.008  0.499 ±0.014  0.531 ±0.012 | 0.533 ±0.011  0.523 ±0.014  0.501 ±0.008  0.513 ±0.008  0.497 ±0.011  0.524 ±0.014 | F_1,17_ = 2.9  p = 0.104  F_1,17.1_ = 4.2  p = 0.055  F_1,17.4_ = 0.4  p = 0.556  F_1,16.9_ = 4.9  *p = 0.040*  F_1,17.2_ = 18.3  p < 0.001  F_1,16.9_ = 19.3  *p < 0.001* | F_1,21.5_ = 2.1  p = 0.159  F_1,21.4_ = 0.009  p = 0.923  F_1,22.1_ = 0.6  p = 0.466  F_1,21.9_ = 1.8  p = 0.190  F_1,21.8_ = 2.9  p = 0.103  F_1,18.5_ = 3.0  p = 0.099 | F_1,16.9_ = 1.9  p = 0.182  F_1,17_ = 0.5  p = 0.497  F_1,17_ = 2.4  p = 0.141  F_1,16.9_ = 2.2  p = 0.154  F_1,17_ = 2.6  p = 0.124  F_1,16.9_ = 1.1  p = 0.313 |
| RD  Superior Cerebellar Peduncle  Right  Left  Middle Cerebellar Peduncle  Right  Left  Inferior Cerebellar Peduncle  Right  Left | 0,000870  ±0,000028  0,000873  ±0,000033  0,000548  ±0,000018  0,000572  ±0,000017  0,000803  ±0,000031  0,001025  ±0,000022 | 0,000886  ±0,000025  0,000934  ±0,000031  0,000544  ±0,000014  0,000580  ±0,000014  0,000875  ±0,000027  0,001077  ±0,000023 | 0,000756  ±0,000027  0,000717  ±0,000032  0,000479  ±0,000017  0,000481  ±0,000017  0,000607  ±0,000030  0,000852  ±0,000022 | 0,000722  ±0,000022  0,000719  ±0,000028  0,000483  ±0,000012  0,000479  ±0,000012  0,000629  ±0,000025  0,000896  ±0,000021 | F_1,16.6_ = 16.1  *p < 0.001*  F_1,17.1_ = 21.1  *p < 0.001*  F_1,17.3_ = 14.1  p = 0.002  F_1,17.4_ = 27.3  *p < 0.001*  F_1,17.3_ = 33.1  *p < 0.001*  F_1,17_ = 38.4  *p < 0.001* | F_1,20.6_ = 0.4  p = 0.553  F_1,19.4_ = 2.4  p = 0.141  F_1,17.9_ = 0.001  p = 0.978  F_1,18.3_ = 0.1  p = 0.790  F_1,20.3_ = 8.4  *p = 0.009*  F_1,19.5_ = 11.2  p = 0.003 | F_1,17_ = 3.3  p = 0.086  F_1,17_ = 2.1  p = 0.165  F_1,17_ = 0.1  p = 0.751  F_1,17_ = 0.2  p = 0.681  F_1,17_ = 2.7  p = 0.121  F_1,17_ = 0.1  p = 0.790 |
| MD  Superior Cerebellar Peduncle  Right  Left  Middle Cerebellar Peduncle  Right  Left  Inferior Cerebellar Peduncle  Right  Left | 0,001209  ±0,000025  0,001200  ±0,000031  0,000776  ±0,000019  0,000799  ±0,000019  0,001047  ±0,000033  0,001025  ±0,000022 | 0,001221  ±0,000025  0,001261  ±0,000029  0,000781  ±0,000014  0,000822  ±0,000016  0,001114  ±0,000029  0,001077  ±0,000023 | 0,001088  ±0,000025  0,001040  ±0,000029  0,000683  ±0,000018  0,000696  ±0,000019  0,000857  ±0,000032  0,000852  ±0,000022 | 0,001056  ±0,000022  0,001046  ±0,000027  0,000687  ±0,000013  0,000694  ±0,000015  0,000885  ±0,000026  0,000896  ±0,000021 | F_1,16.9_ = 20.1  *p < 0.001*  F_1,17.1_ = 25.4  *p < 0.001*  F_1,17.4_ = 25.7  *p < 0.001*  F_1,17.2_ = 29.4  *p < 0.001*  F_1,17.2_ = 28.9  *p < 0.001*  F_1,17.1_ = 38.4  *p < 0.001* | F_1,19.7_ = 0.4  p = 0.546  F_1,18.9_ = 2.4  p = 0.137  F_1,17.9_ = 0.1  p = 0.754  F_1,18.4_ = 0.7  p = 0.426  F_1,19.2_ = 5.4  *p = 0.031*  F_1,19.5_ = 11.2  *p = 0.003* | F_1,16.9_ = 2.2  p = 0.160  F_1,17_ = 1.7  p = 0.205  F_1,17_ = 0.002  p = 0.964  F_1,16.9_ = 0.9  p = 0.365  F_1,17_ = 0.9  p = 0.335  F_1,17_ = 0.1  p = 0.790 |

**Table S6**. Standardized response means of clinical and neuroimaging changes in the *RFC1* cohort.

|  | Mean Difference  (FU-BL) ±SD | SRM |
| --- | --- | --- |
| SARA score | 4.2 ±5.9 | 0.7 |
| Brainstem volume (mm³) | -604.5 ±174.2 | -3.5 |
| Right thalamus (mm³) | -326.9 ±127.7 | -2.6 |
| Left hippocampus (mm³) | -185 ±89.4 | -2.1 |
| Left cerebellar lobule VI (mm³) | -182.9 ±167.3 | -1.1 |
| Left cerebellar lobule X (mm³) | -20.9 ±29.9 | -0.7 |
| Spinal CSA C1 (mm²) | -1.3 ±1.8 | -0.7 |
| Spinal CSA C2 (mm²) | -1.1 ±1.6 | -0.7 |

FU: Follow-up. BL: Baseline. SRM: Standardized response mean. SARA: Scale of Assessment and Rating of Ataxia. CSA: Cross-sectional area.


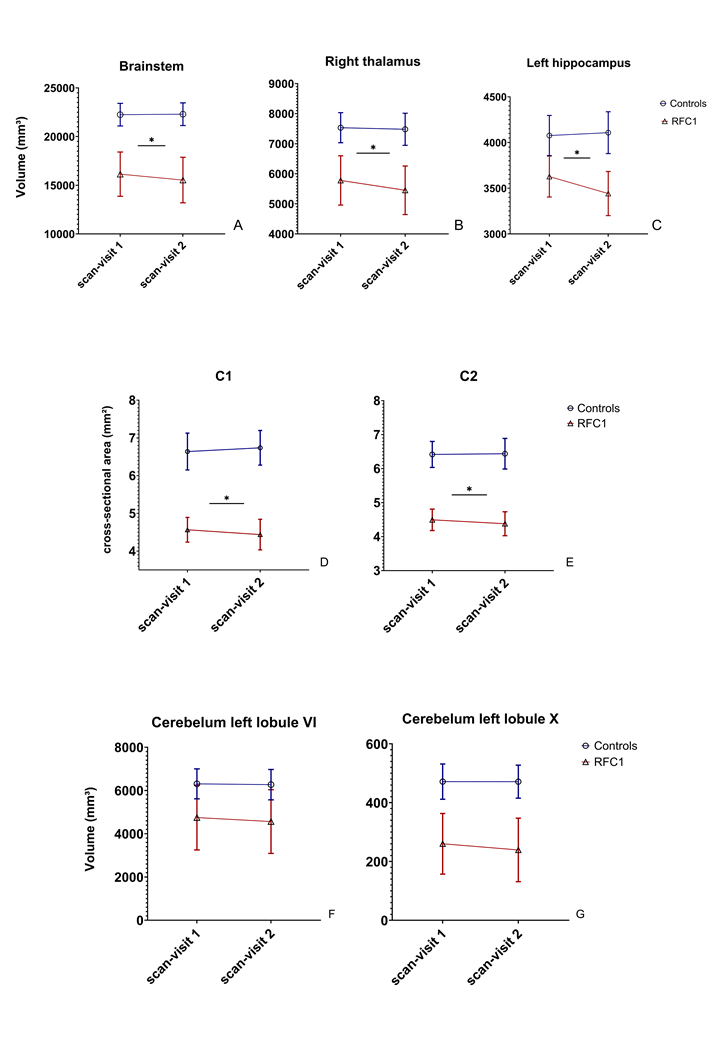


**Figure S2**. Neuroimaging parameters with significant longitudinal changes in RFC1-related disorders *vs* healthy controls. A, brainstem volumetry. B, right thalamus volumetry. A, left hippocampus volumetry. D, spinal cord cross-sectional area at C1 level. E, spinal cord cross-sectional area at C2 level. F, left cerebellar VI lobule volumetry. G, left X cerebellar lobule volumetry. Asterisk indicates significant results in the analysis of simple effects.

**Table S7**. Annualized Volumetric Changes in Brain Regions for RFC1 and Healthy Controls.

| **Region** | **RFC1**  **(mm³/year)** | | | **HC**  **(mm³/year)** | |
| --- | --- | --- | --- | --- | --- |
|  | Median (IQR) | Mean ±SD | Median (IQR) | | Mean ±SD |
| Brainstem | -203.3(87) | -227.4±50 | -40.7(121) | | -34.6±144 |
| Right Thalamus | -195.8(117) | -178.8±75 | -30.7(69) | | -21.8±57 |
| Left Hippocampus | -97.9(85) | -102.8±52 | +28.8(74) | | +26.8±41 |

HC: Healthy Controls. IQR: Interquartile Range. SD: Standard Deviation.
